# A Rapid Method for Determining the Oxidative Stability of Oils Suitable for Breeder Size Samples

**DOI:** 10.1007/s11746-013-2240-1

**Published:** 2013-04-21

**Authors:** R. Przybylski, J. Wu, N. A. M. Eskin

**Affiliations:** 1 Department of Chemistry and Biochemistry, University of Lethbridge, Lethbridge, AB T1K 3M4 Canada; 2 Department of Human Nutritional Sciences, University of Manitoba, Winnipeg, MB R3T 2N2 Canada

**Keywords:** TLC-FID, Polar material, Oxidative stability, Oxidation

## Abstract

A method utilizing thin-layer chromatography with a flame ionization detector (TLC-FID) was developed for assessing the stability of breeder’s oil seed samples based on the formation of polar compounds. The results showed a linear relationship between peroxide value (PV) and the content of polar material in the oxidized oil. Oil samples oxidized very readily on chromarods, even at low temperature, which is a particular advantage for antioxidant screening. At 45 °C, the oil oxidation rate was relatively low, but the relationship between the content of polar material and reaction time was linear. At 65 °C, if the content of polar material was below 50 %, the above relationship was still linear. At different temperatures, the action of tocopherol appeared to vary slightly. For example, at 65 °C, the oxidative stability of the oil sample was determined by the content of tocopherol, especially γ-tocopherol. At 45 and 55 °C, the oxidative stability was determined by both the content of tocopherol and polyunsaturated fatty acids. Of the tocopherol isomers, γ-tocopherol exhibited the highest antioxidant potency, consistent with the published literature. These results suggest that chromarods provide good media for monitoring oil oxidation for antioxidant screening. A particular advantage is the use of very small oil samples, usually 1–2 μL, and the ability to analyze multiple samples at the same time.

## Introduction

Lipids containing polyunsaturated fatty acids readily oxidize in the presence of oxygen, making oxidative rancidity one of the most critical factors affecting the shelf**-**life of foods. During autoxidation, a number of polar compounds may be produced from the initially formed hydroperoxides [1, 2]. The content of these polar materials can be determined by thin layer chromatography with a flame-ionization detector (TLC-FID).

TLC-FID uses permanent rod-shaped layers, whose mechanical and chemical properties permit detection of the separated components with a flame ionization detector. Over the last 20 years, the TLC-FID method has become an important quantitative method, comparable to GLC or HPLC, for separating chemical substances in medicine and biology as well as in lipid chemistry including characterization of crude oils [3]. Because of its high sample throughput, it is particularly useful for quantitative separations of substances that cannot be analyzed by gas chromatography because of their low volatility or because of the irreversible adsorption of components on the column. Most applications of TLC-FID in the food industry and related fields are focused on analyzing fats and their derivatives, i.e. acylglycerols, fatty acids, and phospholipids [4–9]. Furthermore, it can be used to quantitatively determine the content of polar components in frying oils. Compared to column chromatography with silica gel and Sep-Pak cartridges (silica gel), TLC-FID analysis does not require large quantities of solvent and samples. The results obtained by TLC-FID correlate well with those obtained by column chromatography and are a little higher than those by Sep-Pak cartridges [10]. It appears that the TLC-FID method can be used to indirectly determine the peroxide value (PV) of oxidized oil.

Like silica gel in thin layer chromatography, the sintered mixture of glass and silica gel adsorbent has a large surface area. For example, silicate glass Vycor containing 96 % silicon dioxide has a specific surface area of 150 m^2^/g [11]. The ratio of surface area to volume is very high, so that the oxidation rate on the chromarod will be high. Compared with the Schaal oven test, it should take much less time to reach the same PV. In addition, assessment of oxidative stability can be done at low temperatures, so that the results obtained can be used to assess the actual oxidative stability of oil or food**-**containing oil/fat.

Generally, there are two approaches for determining potential oxidative stability [2, 12]. The first is to evaluate the induction period observed before significant production of peroxides (or secondary products) begins. The induction period is highly dependent on the conditions of the oxidation experiment. As such, the oxidation experiment must be carried out according to well-established protocols. These methods include the Schaal oven test, the Swift test or active oxygen method (AOM), and its automated forms such as the Rancimat apparatus or oil stability index (OSI) method. The OSI values are generally well correlated to the corresponding AOM values if the PV is 100 or greater. The method is automated and thus easier to use than the AOM. However, it is time-consuming and suffers from the common problem that large errors can be incurred from small variations in the airflow rate.

The other principal option for assessing potential oxidative stability is monitoring the rate of peroxides formation or oxygen consumption. The sample is sealed under air or oxygen and stored at a constant temperature. The oxygen concentration in the headspace is monitored by periodically withdrawing small samples through sealed septa affixed to the container and analyzing oxygen by gas chromatography. It includes the Sylvester test and the automated version FIRA-Astell apparatus and Oxidograph.

The fundamental problem with all of these methods is that lipid stability is determined at a fixed temperature, usually far above the ambient, because it takes an unacceptable long time to obtain meaningful measurements. It is known that the values for oxidative stability thus obtained cannot always be used for quantitative and even semi-quantitative estimation of the important storage characteristics of lipids at ambient temperature.

Consequently, it is important to find a new approach that can be used to assess the oxidative stability of lipids at ambient temperature within a reasonable time period. This paper reports the use of TLC-FID to assess the oxidative stability of lipids at low temperature.

## Materials and Methods

### Chemicals

All the solvents were of chromatographic purity and used directly without purification. α-Tocopherol and γ-tocopherol were purchased from Sigma (St. Louis, MO, USA).

### Oil Samples

Commercially refined, bleached and deodorized canola oil (CAN) without antioxidants added was obtained from Richardson Oilseeds (Lethbridge, Canada) while flaxseed oil (F) was purchase from a local health food store. Mid-oleic sunflower oil (MSUN) was obtained from ADM (Decatur, IL, USA).

### Accelerated Storage

Oil storage experiments were conducted in an oven according to the Schaal Oven test. Oils were stored in open clear glass jars keeping ratio between surface areas to volume at 1 at 65 °C. At fixed intervals oil samples were withdrawn, and PV was determined according to the AOCS official method Cd 8-53 [13].

### Oil Frying Experiment

Oil samples with and without added α- or γ-tocopherol were used for frying experiments. The simulated frying was conducted at a frying temperature of 180 ± 5 °C with stirring. The frying time was 8 h a day, and the total time was 40 h. The oil sample was withdrawn after each day of heating for determination of polar materials and tocopherols.

### Determination of Polar Material

**Fig. 1:**
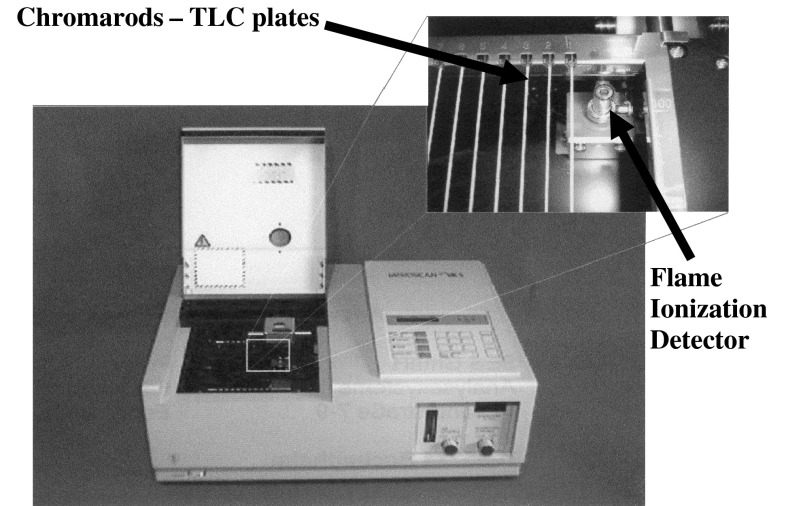
The Iatroscan–TLC-FID system. TLC plates in the form of chromarods and FID detector are shown

The polar material was separated on a sintered silica gel on quartz rods and detected with a flame ionization detector (FID) (Iatroscan TH-10IV; Iatron, Japan; Fig. 1). The hydrogen and airflow rates were maintained at 185 mL/min and 2 L/min, respectively. Chromarod SIII (Iatron Laboratories, Tokyo, Japan) was used for separation. On each chromarod, 1.6 μL of 200 mg oil dissolved in 1.8 mL chloroform was applied using a microsyringe. The rods were developed in a hexane-diethyl ether-acetic acid (92:8:1) solution for 25 min. After development, the rods were dried in an oven at 105 °C for 2 min and then scanned with FID.

The amount of polar compounds was also assessed by the gravimetric reference method following the AOCS method Cd 20-91 with the Schulte modification [13, 14].

### Oxidation on Chromarods

Oil samples were applied to the rods and stored in oven at 45, 55, and 65 °C for 30 min. The rods were then developed in the solvent system and the amount of polar material determined as previously described. Different oxidation times and temperatures were chosen and the content of polar material determined to compare the oxidative stability of the different oils. For quantification of polar material, an external calibration method was used applying different amounts of polar components. The amount of polar components was also measured according to AOCS Cd 20-91 [13].

### Determination of Tocopherols

Tocopherol contents were determined by normal HPLC, using a Shimadzu LC-10AD apparatus (Shimadzu, Tokyo, Japan) equipped with a fluorescence detector RF-10AXL set up for excitation at 290 nm and emission at 335 nm. A Prodigy 5-μm silica column (250 × 3.2 mm; Phenomenex, Torrance, CA, USA) was used, and elution was done with *tert*-butyl methyl ether/hexane (5:95, v/v) at a flow rate of 0.8 mL/min. For quantification, each tocopherol isomer was calibrated individually.

### Statistical Analysis

Samples from three repetitions of each protocol were collected and analyzed in triplicate. Data are presented as mean value ± SD. Data was analyzed by single factor analysis of variance (ANOVA) and regression analyses using SPSS 10.0 software (SPSS, Chicago, IL, USA). Statistically significant differences between means were determined by Duncan’s multiple range tests at *P* ≤ 0.05.

## Results and Discussion

### Determination of PV by TLC-FID

**Fig. 2:**
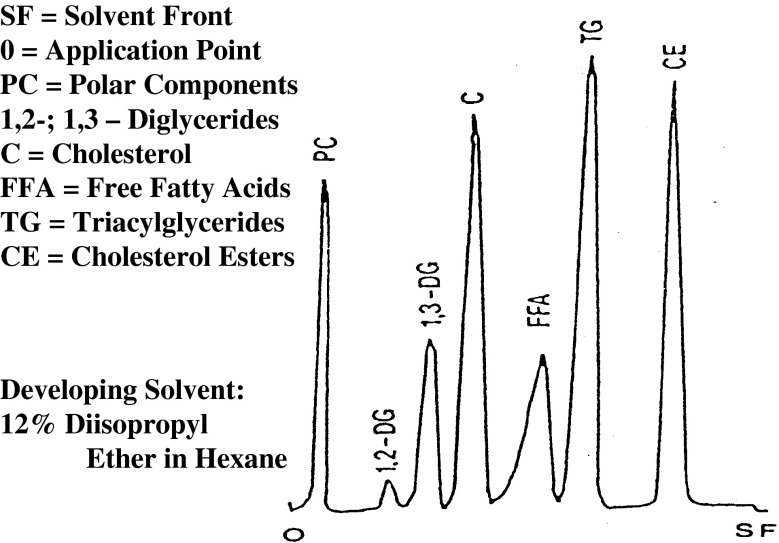
TLC-FID chromatogram of lipid components

**Fig. 3:**
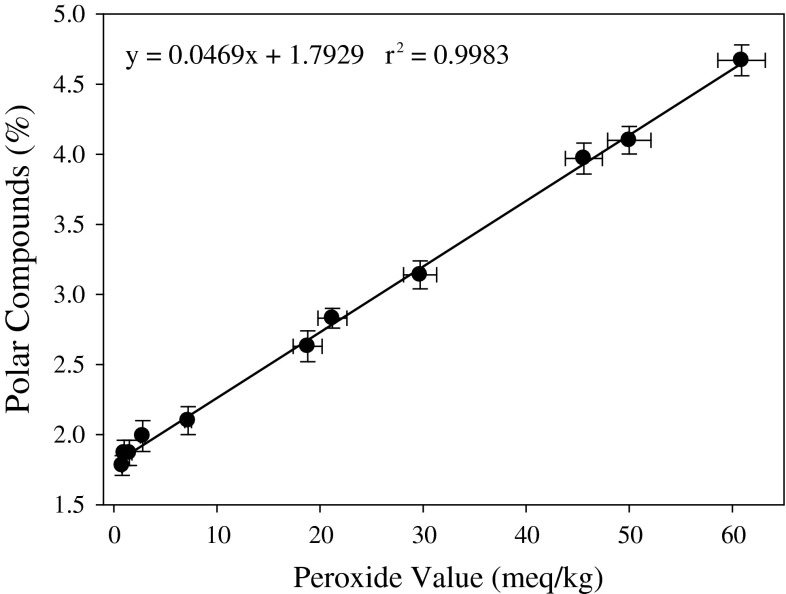
Relationship between peroxide value and polar components (measured by TLC-FID) in canola oil stored at 65 °C. *Line* represents regression

There are many analytical methods used for measuring oxidative deterioration in fats and oils, foods, and biological systems. These methods can be categorized into four groups based on what they measure: the absorption of oxygen, the loss of initial substrate, the formation of hydroperoxides as primary oxidation products, or the formation of secondary oxidation products from hydroperoxides decomposition [14–17]. These methods only determine the total amount of hydroperoxides and include chemical methods based on redox reactions, methods based on enzymatic reactions, and measurements of physical properties such as UV spectroscopy for detection of conjugated dienes: Fourier transformed infrared (FTIR) spectroscopy for changes in structure of fat molecules and size exclusion chromatography [17]. It has been previously reported that TLC-FID can be used for the determination of polar materials in the frying oil [10]. A typical separation of lipid classes using TLC-FID is presented in Fig. 2. Baseline separation of most classes of lipids can also be achieved, where polar compounds are nicely isolated from others. Hydroperoxides can also be considered to be polar material, so that TLC-FID may be used for determining hydroperoxides in the oil similar to the standard methods used for their assessment. Figure 3 shows the correlation between peroxide value and the amount of polar compounds for canola oil stored at 65 °C at different stages of oxidative degradation. Using TLC-FID, high accuracy determination of polar compounds was achieved which is directly related to the peroxide value. In order to get reproducible estimates of the hydroperoxide content in the oil at the lower level, a relatively larger amount of oil sample, 160 μg, was applied to the chromarods. At 1 % of polar material in the applied sample, the estimated amount of hydroperoxides will be at 1.6 μg, this value is close to the level of detection for TLC-FID, but reproducible [11]. According to the literature, a coefficient of variation (CV) for the hydroperoxides assessment for standard method is about 5 % at the lower level of these compounds, and this value is lower when the amount of these compounds is higher [3]. A similar CV was obtained for the assessment of polar compounds by TLC-FID where a much lower amount of sample is required. Assessing the polar material can also be used for the indirect determination of PV. A real advantage of the TLC-FID method is that the amount of sample required for assessment is at the microgram level compared to grams of oil needed for the classical PV method. Consequently, the small amount of sample and short time required for assessment using the TLC-FID procedure makes it particularly useful for assessing oil stability when the amount of oil is limited.

These results suggest that measurement of polar material is a better index of oxidative degradation of oil. TLC-FID methodology is highly operator-dependent, so that, to ensure reliable results are obtained multiple measurements, should be conducted. This is facilitated using ten chromarods at the same time, by running concurrently multiples of the same sample under the same conditions including standards for comparison. These results suggest that TLC-FID can be used for the indirect determination of the PV of oil in addition to measuring the polar material content of frying oil.

### Oxidation on Chromarods

The conventional methods for determining oil stability require large quantities of oil samples, and take a long time to reach the endpoint. Furthermore, the oxidation of oil is conducted at a higher temperature to accelerate the oxidation rate and shorten the time for oxidation to develop. Obviously, the oxidation process at high temperature is different from that at room temperature, so that the results obtained cannot be directly extrapolated to predict the shelf life of oil or oil-containing products. It is generally accepted that the mechanism of oxidation changes significantly at elevated temperatures, so that, of all the accelerated stability tests, the Schaal oven test conducted between 60 and 70 °C has the fewest problems and most closely reflects ambient room changes [18]. While testing the stability of oils at ambient temperature would be ideal for determining real storage conditions, it is far too slow to be of practical value [18]. The proposed TLC-FID method, however, permits testing oxidation of the oil at lower temperatures because of the large surface area of silica on the chromarod. When applied on its surface, the oil will have a very large surface area to volume ratio. This will facilitate a very high oxidation rate due to this surface area exposed to oxygen, even at lower temperatures. The polar materials formed during oil oxidation are readily separated by developing the chromarods in the solvent system described and then measured by the FID detector, a universal and highly sensitive detector for organic compounds.

**Fig. 4:**
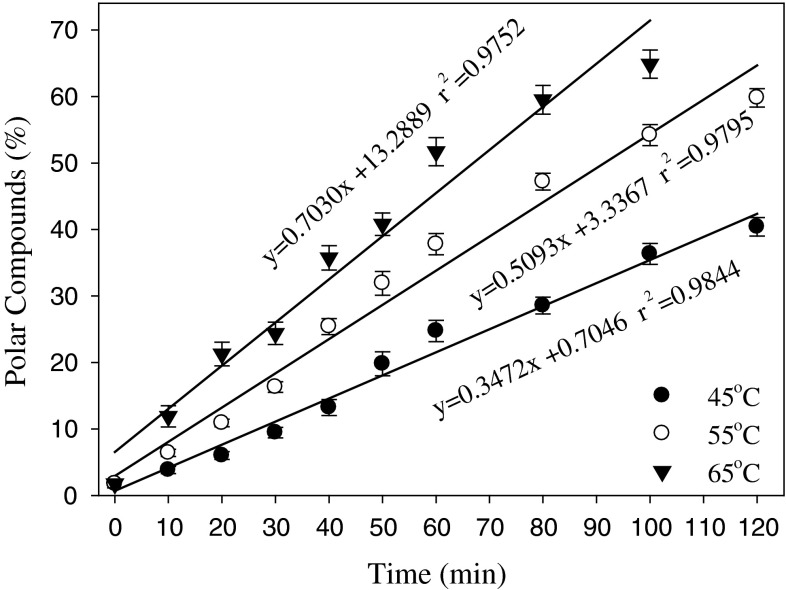
Effect of heating time on the amount of polar compounds as measured by oxidation on chromarods. Oxidation was performed at three different temperatures

Figure 4 illustrates the relationship between the content of polar material, oxidation time, and temperature for canola oil. At 45 and 55 °C, the relationship between the amount of polar material and oxidation time is linear, with a coefficient of determination at 0.9844 and 0.9795, respectively. This is quite different from the oil oxidation curves determined by conventional methods at the same temperature range where the induction period is clearly visible and used for stability assessment. In the case of oxidation on chromarods where oil is exposed over a large surface area to oxygen, oxidation is running at a much faster rate with an extremely short induction period. A large portion of the double bonds in the polyunsaturated fatty acids come in contact with oxygen and react quickly even at low oxidation temperatures. The larger surface area appears to have a greater impact on the oxidation rate of oil than elevated temperatures. The CV for all determinations for polar material was below 5 %, indicating high reproducibility of the applied TLC-FID procedure including oxidation on chromarods. Increasing the temperature to 55 °C resulted in a 50 % increase in oxidation with a faster accumulation of polar material over a time period. This would allow more samples to be assessed in the same period of time, so that the oxidative stability of 50–75 samples could be evaluated at the higher temperature during the normal working day. The increased capacity for sample assessment using this procedure would meet the need of breeders to evaluate individual lines for data about oil stability providing practical information for crop selection.

By comparison, the curve of polar compounds formed during oxidation at 65 °C was not quite linear compared to the other temperatures (Fig. 4). At the beginning of oxidation, the curve is close to linear, but after 45 min of oxidation the curve starts to plateau. In addition, this curve has coefficient of determination at 0.9752, which is lower compared to other two temperatures. This behavior can be attributed to the exhaustion of the substrate available for oxidation after 40 min of heating. The above results indicate that oxidation at 65 °C, commonly used for accelerated storage in the Schaal oven test, is faster although changes in polar material are not linear and can complicate assessment of oxidative stability of the oil based on the formation of polar compounds.

According to the relationship between the content of polar material and PV for canola oil (Fig. 3), the amount of the former will reach a level equivalent to PV = 50 when oxidized at 45 °C for 20–25 min, whereas during heating at 55 °C, the time to reach the same PV value will be 10–15 min. Using higher temperature, we can shorten analysis time and run more samples per time unit. This is particularly important when the method is applied for assessment of breeder lines. However, if the oil samples contain native or added antioxidants, or the fatty acid forming triglycerides are more saturated, the assessment of oxidative stability may need to be conducted at a higher temperature or by extending the time of heating.

### Stability of Oils

**Fig. 5:**
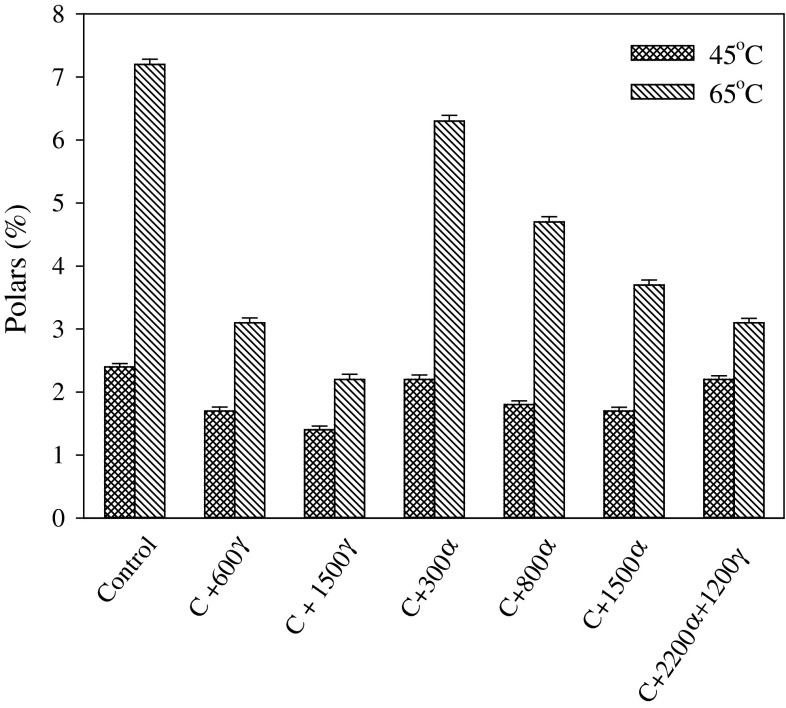
Stability of canola oil fortified with different tocopherols as measured by TLC-FID system. α and γ represent tocopherol isomers added at different amounts

In order to verify the validity of the above method for assessing oxidative stability, oil samples with or without antioxidant added were analyzed by TLC-FID. The differences in oxidative stability between oil samples containing different tocopherol concentrations are shown in Fig. 5. Canola (CAN) oil without added antioxidant had the lowest oxidative stability and exhibited the highest amount of polar components formed. As the concentration of γ-tocopherol increased, the amount of polars decreased significantly showing effective protection. Thus, oil oxidative stability increased with a corresponding increase in γ-tocopherol content. Fortifying CAN oil with α-tocopherol also improved oxidative stability, but was less effective compared to the gamma isomer (Fig. 5). This suggests that, at higher concentrations, α-tocopherol had an adverse effect on the oxidative stability of oil compared to γ-tocopherol the gamma isomer. These results agree with finding from other publications showing that α-tocopherol can act as a prooxidant or loses efficacy when present in oil at high concentrations [17–20]. The results for CAN with 1,500 μg/g of γ-tocopherol showed stronger antioxidant potency compared to CAN with the same amount of α-tocopherol (Fig. 5). When oxidation was carried out at 65 °C, a similar pattern of changes was observed showing that γ-tocopherol was more effective in protecting oil against oxidative degradation than α-tocopherol (Fig. 5). More pronounced differences in antioxidative potency of α-tocopherol were observed when the amount of it increased (Fig. 5). This suggests that, with the increase in oxidation rate at higher temperature, α-tocopherol acts as an antioxidant when present at high concentration, while, at the lower oxidation rate at a lower temperature, too much α-tocopherol will be oxidized so that the tocopherol radicals may act as initiators of oil degradation. These results confirm that γ-tocopherol has stronger antioxidant potency compared to α-tocopherol.

### Polar Components Assessment

**Fig. 6:**
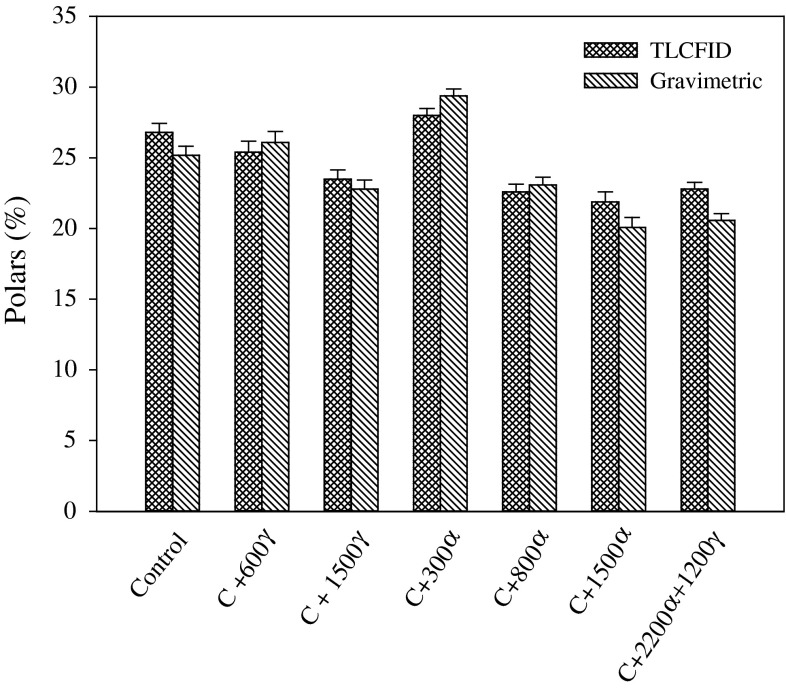
Formation of polar compounds during heating canola oil with added tocopherols as measured by TLC-FID and standard gravimetric method. α and γ represent tocopherol isomers added at different amounts

Oils with added antioxidants and control oil were heated for 8 h at frying temperature with the amount of polar compounds measured by the gravimetric method and compared to the TLC-FID method (Fig. 6). For all oils, the amounts of polars measured by both methods were almost identical; differences were within analytical error of applied procedures (Fig. 6). Similar results were also observed for mid-oleic sunflower (MSUN) oil in the presence of different amounts of added antioxidants (results not included).

### Tocopherol Stability

After 1 day of frying, the degradation of tocopherols in canola oil ranged from 50 to 90 %. Of the tocopherol isomers, the γ isomer disappeared at the faster rate compared to α-tocopherol (Table 1). These results provide further evidence that heating oil without food at frying temperatures is more destructive. This is probably due to the oil being constantly exposed to oxygen, whereas during frying, water vapor from the food forms protective barrier [19].

**Table 1:** Effect of frying on tocopherol content of canola and mid-oleic sunflower oils

Sample	Frying time (h)	Tocopherols (ppm)		
		α	γ	Total
CAN	0	238	460	698
CAN + 600γ	0	317	894	1,211
CAN + 1,500γ	0	404	1,291	1,695
CAN + 300α	0	600	440	1,040
CAN + 800α	0	1,220	456	1,676
CAN + 1,500α	0	1,548	434	1,982
CAN + 2,200α + 1,200γ	0	2,630	1,665	4,431
CAN	8	90	8	98
CAN + 600γ	8	170	42	212
CAN + 1,500γ	8	217	32	249
CAN + 300α	8	101	11	112
CAN + 800α	8	505	34	539
CAN + 1,500α	8	835	78	913
CAN + 2,200α + 1,200γ	8	1,915	584	2,499
MSUN	0	720	20	740
MSUN + 1 % F	0	731	27	758
MSUN + 2.6 % F	0	723	61	784
MSUN + 5 % F	0	690	85	775
MSUN + 500γ	0	824	563	1,387
MSUN + 500γ + 2.5 % F	0	770	531	1,301
MSUN	8	140	0	140
MSUN + 1 % F	8	193	0	193
MSUN + 2.6 % F	8	179	0	179
MSUN + 5 % F	8	236	5	241
MSUN + 500γ	8	323	67	390
MSUN + 500γ + 2.5 % F	8	304	31	335

*CAN* and *MSUN* canola and mid-oleic sunflower oils, respectively, *F* flaxseed oil added

**Fig. 7:**
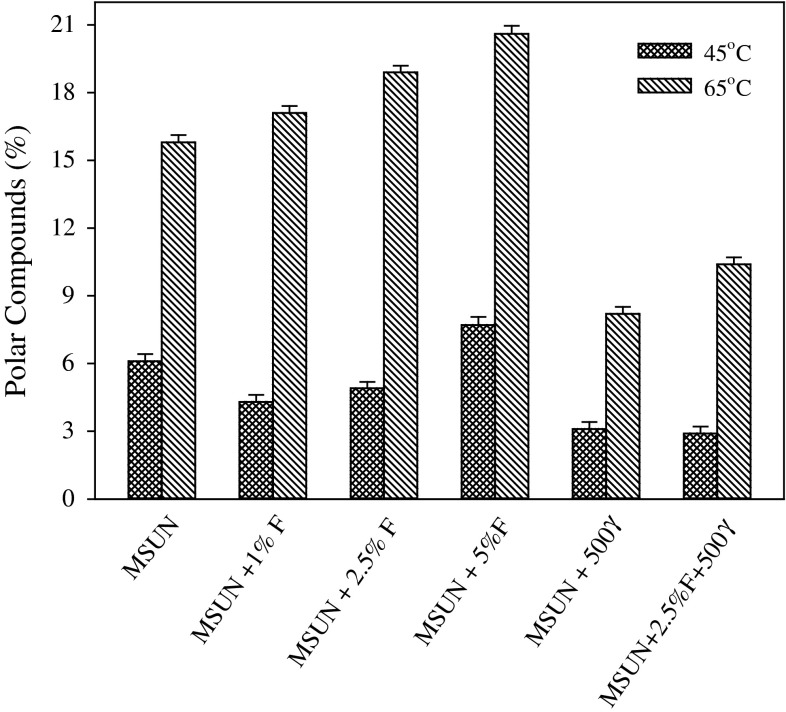
Oxidative stability of sunflower oil fortified with flaxseed oil and antioxidants as measured by TLC-FID system. γ represents tocopherol isomer added at different amounts

Mid-oleic sunflower oil (MSUN) containing about 60 % of oleic acid, was fortified with flaxseed oil and tocopherol. When flaxseed oil was added, the content of PUFA was increased, which would be expected to affect the oxidative stability of the oil blend. Oxidative stability of MSUN blends was assessed using TLC-FID with the results shown in Fig. 7. Far more pronounced differences were evident when oils were oxidized at 65 °C, with an increase in flaxseed oil causing a decrease in oxidative stability. However, a different pattern of oxidative stability was observed for the same oils oxidized at 45 °C (Fig. 7). Addition of 500 γ-tocopherol increased oxidative stability compared to MSUN, while MSUN containing 2.5 % flaxseed oil and 500 μg/g γ-tocopherol also showed a significant improvement in oxidative stability as measured by the lower amount of polar components formed.

The addition of both 1 and 2.5 % flaxseed oil to MSUN increased its oxidative stability, as shown by the lower amount of polar components (Fig. 7). However, further addition of flaxseed oil to 5.0 % decreased its oxidative stability, in spite of increasing the content of γ-tocopherol. The presence of endogenous α-tocopherol alone cannot determine the oxidative stability of oil. These are contrary to the results obtained at 65 °C which suggests that tocopherol acts differently on oil samples with different PUFA content. Higher oxidation rates were achieved at 65 °C, indicating that the content of PUFAs appeared to have little effect on the oxidative stability of oil sample. The lower oxidation rates observed at 45 °C, however, suggested that the content of PUFAs and tocopherols both determined the oxidative stability of the oil sample. At both temperatures, the presence of higher amounts of γ-tocopherol increased oxidative stability of oils, indicating better antioxidative efficiency by this isomer compared to corresponding α-isomer.

## Conclusion

The above results further confirm that oxidative stability of oils is determined by the content of both PUFAs and tocopherols [21]. When tocopherols were applied with oil on chromarods, they can still protect oil from oxidation. Chromarods proved to be an excellent system for studying oxidative stability, as large surface area to volume facilitates a high oxidation rate. The TLC-FID method has special application for screening and assessing antioxidants from natural sources like plants, particularly when only small amounts of oil samples are available. An additional advantage is the ability of TLC-FID to assess a large number of samples in a relatively short period of time. A further advantage is the use of microgram amounts of oil samples to assess potential oxidative stability of the particular oil or fat.
